# REAC technology as optimizer of stallion spermatozoa liquid storage

**DOI:** 10.1186/s12958-017-0229-6

**Published:** 2017-02-08

**Authors:** Fiammetta Berlinguer, Valeria Pasciu, Sara Succu, Ignazio Cossu, Sabrina Caggiu, Daniela Addis, Alessandro Castagna, Vania Fontani, Salvatore Rinaldi, Eraldo Sanna Passino

**Affiliations:** 1 Department of Veterinary Medicine, University of Sassari, Viale Vienna 43/B, 07100 Sassari, Italy; 2 AGRIS, Department of Research for Equine Reproduction, Ozieri, Sassari Italy; 3 Departments of Regenerative Medicine, Rinaldi Fontani Institute, Viale Belfiore 43, 50144 Florence, Italy; 4 Research Department, Rinaldi Fontani Foundation, Viale Belfiore 43, 50144 Florence, Italy

## Abstract

**Background:**

REAC technology (acronym for Radio Electric Asymmetric Conveyor) is a technology platform for neuro and bio modulation. It has already proven to optimize the ions fluxes at the molecular level and the molecular mechanisms driving cellular asymmetry and polarization.

**Methods:**

This study was designed to verify whether this technology could extend spermatozoa life-span during liquid storage, while preserving their functions, DNA integrity and oxidative status. At 0, 24, 48, and 72 h. of storage at 4 °C, a battery of analyses was performed to assess spermatozoa viability, motility parameters, acrosome status, and DNA integrity during REAC treatment. Spermatozoa oxidative status was assessed by determining lipid peroxidation, the activity of superoxide dismutase (SOD), and the total antioxidant capacity.

**Results:**

During liquid storage REAC treated spermatozoa, while not showing an increased viability nor motility compared to untreated ones, had a higher acrosome (*p* > 0.001) and DNA integrity (*p* > 0.01). Moreover, the analysis of the oxidative status indicated that the mean activity of the intracellular superoxide dismutase (SOD) was significantly higher in REAC treated spermatozoa compared to untreated controls (*p* < 0.05), while the intracellular concentration of malondialdehyde (MDA), an end product of lipid peroxidation, at the end of the REAC treatment was higher in untreated controls (*p* > 0.05). The REAC efficacy on spermatozoa oxidative status was also evidenced by the higher trolox equivalent antioxidant capacity (TEAC) found in both the cellular extract (*p* < 0.05) and the storage media of REAC treated spermatozoa compared to untreated controls (*p* < 0.0001).

**Conclusion:**

The present study demonstrated that REAC treatment during liquid storage preserves spermatozoa acrosome membrane and DNA integrity, likely due to the enhancement of sperm antioxidant defenses. These results open new perspective about the extending of spermatozoa functions in vitro and the clinical management of male infertility.

**Electronic supplementary material:**

The online version of this article (doi:10.1186/s12958-017-0229-6) contains supplementary material, which is available to authorized users.

## Background

Radio Electric Asymmetric Conveyer Technology for therapeutic use (REAC) is a recent technology whose aim is to optimize the ion fluxes at the molecular level, and to concentrate all the micro currents produced by these ion fluxes through the asymmetric conveyer-probe to optimize the molecular mechanisms driving cellular asymmetry and polarization. In fact, it is able to properly asymmetrically convey the ion currents, “re-setting” the altered bioelectric fields and therefore recovering the correct cell electro-metabolic activity which drives the migration, proliferation and differentiation of a host of cell types and promotes reparative and regenerative process of tissues [1–8].

Sperm cells are polarized cells [9]. Development, survival, and effectiveness of sperm are closely linked to the phenomena of cell polarity. On these bases, we checked whether REAC technology is effective in extending and maintaining sperm functions and DNA integrity during liquid storage at 4 °C. In fact, sperm cryopreservation and sperm liquid storage by short-term refrigeration at +4 °C lead to a significant decline in sperm quality [10]. In particular, during sperm liquid storage, we observe a deterioration of sperm functions also followed by DNA damage [11].

Oxidative stress has been proposed to be a major factor involved in sub lethal cryodamage of sperm in many species, including horses [12, 13]. After semen dilution and cryopreservation, there is a significant reduction in the level of spermatozoa antioxidants, thus leading to an enhanced susceptibility of these cells to peroxidative injuries. In addition, semen cryopreservation is associated with increased generation of reactive oxygen species (ROS) [14, 15]. Spermatozoa are vulnerable to oxidative attack because they contain an abundance of polyunsaturated fatty acids that are susceptible to lipid peroxidation. Functionally important proteins and DNA are also subject to oxidative modification and adduction by aldehydes, generated as a consequence of the peroxidative process [16]. Oxidative stress ultimately triggers the intrinsic apoptotic pathway, leading to a rapid loss of motility and cell death. As suggested by Riel et al. [17], the iatrogenic damage caused by semen dilution and cryopreservation may be also due to the presence of ions in the surrounding medium coupled with sperm membrane changes occurring during storage at non-physiological temperatures. Sodium-potassium-dependent ATPase, which regulates proper intracellular concentration of sodium, is highly sensitive to hypothermia. At low temperatures, reduction of its activity impairs proper functioning of the sodium pump, resulting in uptake of sodium ions from extracellular milieu. Sodium ions were shown to be detrimental to the nucleus and DNA [18, 19]. In addition, a perturbation of ion flows can result in alterations of plasma and mitochondrial membranes resting potential and thus of cell functions. Plasma membrane resting potential arises from the combined action of ion channels and ion pumps, and it regulates cell-autonomous properties such as proliferation, differentiation and apoptosis in mature somatic cells as well as stem cells [20]. The loss of mitochondrial membrane potential has been associated with a significant increase in ROS generation [21, 22]. All these phenomena are related to alterations of cell polarity and may be responsible for hampering cell preservation at low temperatures.

Thus, considering the abovementioned REAC treatment effects, this study was designed to test whether this technology may be used to extend spermatozoa lifespan and functions during liquid storage at 4 °C. Since spermatozoa are particularly vulnerable to oxidative attack, the effect of this technology on sperm cell oxidative status during liquid storage was also studied. In the present study, we choose the stallion as experimental model because REAC protocol of Veterinary Neuro Psycho Physical Optimization (VNPPO) in vivo has proven to exert a positive effect in the semen quality of idiopathic subfertile stallions [23].

## Methods

### Chemicals

All chemicals in this study were purchased from Sigma Chemical CO. (St. Louis, MO, USA), unless stated otherwise.

### Description of Radio Electric Asymmetric Conveyer (REAC) Technology

Radio Electric Asymmetric Conveyer Technology (REAC) is a technological platform for bio and neuro modulation. A detailed description of REAC mechanism of action can be found in Maioli 2016 [2]. Briefly, REAC is an asymmetric technology, because a normal electric circuit has two physical poles: one positive and one negative (symmetrical circuit); in the REAC technology, there is only one single physical pole (asymmetrical circuit). This pole becomes the attractor (Asymmetric Conveyer) for the currents induced in the body by the radio frequency emission. This scheme has been developed to create an asymmetric circuit for better interact with the asymmetric mechanism underlying the cell polarity [24], in order to optimize its functions. In fact, REAC technology is able to modulate the current flows existing both at cellular and body level, when these are altered. Another peculiarity of REAC Technology is the low power level used in radio frequency emission. This is necessary to induce current flows of intensity comparable with those of cell polarity. Higher power levels would disturb the adjustment mechanisms of cell polarity. REAC devices use only two radio frequencies (2.4 and 5.8 GHz) because these are the most widely used and permitted at the international level. In this study, we used the biomodulation REAC-VIVSI treatment protocol. The REAC device used in this study was B.E.N.E (ASMED, Florence, Italy).

### Semen source and preparation

The experimental procedures were carried out during horse breeding season (January–July). Semen collection was carried on at the Department of Stallion Reproduction of the Regional Agency for Research in Agriculture (AGRIS Sardegna, Ozieri, Sassari, Italy), while the analytical work was carried on at the laboratories of the Department of Veterinary Medicine of the University of Sassari (UNISS, Sassari, Italy). These facilities meet the requirements of the European Union for Scientific Procedure Establishments. Ejaculates were obtained by artificial vagina from eight adult stallions of different ages (from 12 to 24 years), and different breeds. In particular, stallions were identified with progressive number from 1 to 8 (stallion 1: Thoroughbred; stallions 2, 3, 4, 6: Arabian; stallions 5, 7, 8: Warmblood). The stallions were housed individually in boxes, and fed ad libitum with a diet containing adequate nutrients. At the moment of the study, they had been housed in AGRIS for over 5 years and used exclusively for breeding, and they are currently still housed at the same center. All evaluated stallions had a career in sports flat racing and show jumping.

The eight stallions enrolled in this study were used for breeding, and the frequency in semen collection during the entire breeding season was twice a week. We used for this study two ejaculates from each male, collected 15 days apart from each other. Semen was transported to the AGRIS laboratory within 5 min after collection, and it was immediately processed. Sperm concentration was evaluated by NucleocounterSp - 100 Chemometech. Thereafter, semen was diluted up to 50 ×10^6^ spermatozoa/mL with a commercial media for liquid storage (Kenney's extender, IMV Technologies). Ejaculates from each stallion were kept separated throughout all experimental procedures and, once diluted, they were placed in two separated Falcon tubes and transported to the UNISS laboratory under controlled temperature (4 °C) within 1 h.

Upon arrival, one sample from each stallion was allocated to the treated and untreated group. The experimental groups were placed in two refrigerators at 4 °C and kept there for 72 h. The REAC device was placed in the refrigerator where treated samples were stored, and it was set at 2.4 GHz, and its conveyer electrodes were immersed into the semen liquid storage media.

### Experimental design

In order to evaluate the effects of REAC technology during stallion semen liquid storage, a battery of analyses was performed. The parameters analyzed included different semen molecular and cellular features, measured before and during REAC treatment, such as viability, motility parameters, acrosome status, and DNA integrity. In addition, the oxidative status was assessed by determining lipid peroxidation, the activity of superoxide dismutase (SOD), and the total antioxidant capacity. These analyses were performed on sub-samples of cooled semen collected at 0, 24, 48, and 72 h. after the beginning of the REAC treatment. Each analysis was replicated 3 times.

### Viability and motility parameters assessment

In vitro viability was assessed by eosin-nigrosin stain. Briefly, the eosin–nigrosin solution was prepared as described by Pintado et al. [25]. Briefly, 10 g nigrosin was dissolved in distilled water by boiling, and filtered into a cylinder containing 0.7 g eosin, 7.5 ml of 50 mmol glucose l–1, and 7.5 ml tartrate phosphate buffer (TPB) (50 mM Na2HPO4 l–1, 25 mM KH2PO4 l–1, 77 mM potassium sodium tartrate l–1), and the volume made up to 100 mL. The solution was kept at 5 °C. Staining was carried out by mixing an aliquot of spermatozoa suspended in saline medium with eosin–nigrosin solution (1:3 dilution) for 30 s before preparing a smear and drying on a warm plate at 37 °C. At least 200 cells were counted for each slide. Sperm motility parameters were assessed using a computer-assisted sperm analysis (CASA) system (Sperm Class Analyser, S.C.A. v 3.2.0, Microptic S.L., Barcelona, Spain) with setting of 25 frames acquired to avoid sperm track overlapping, minimum contrast 10, minimum velocity of average path 30 μm/s, progressive motility > 80% straightness. This system has a specific set-up for stallion sperm evaluation. In particular, it was set up as follows: minimum contrast – 70; low and high static size gates – 0.6–4.32; low and high intensity gates – 0.20–1.92; low and high elongation gates 7–91; default cell size – 10 pixels; default cell intensity −80. For each sample, 5 μL subsample of sperm suspension was loaded into a pre-warmed analysis chamber with a depth of 10 μm (Makler Counting chamber, Sefi-Medical Instruments ltd., Biosigma S.r.l., Italy) and a minimum of 500 sperms per subsample were analyzed in at least four different microscopic fields. Sperm motility was assessed at 37 °C at 40 × using a phase contrast microscope. The parameters evaluated included: percentage of progressive motile spermatozoa (PM); percentage of rapid spermatozoa (rapid); average path velocity (VAP, mm/s; the average velocity of the smoothed cell path); curvilinear velocity (VCL, mm/s; the average velocity measured over the actual point to point track followed by the cell); straight-line velocity (VSL, mm/s; the average velocity measured in a straight line from the beginning to the end of the track); linearity index (LIN, %; the average value of the ratio VSL/VCL); straightness index (STR, %; the average value of the ratio VSL/VAP); amplitude of lateral head displacement (ALH, mm; the mean width of the head oscillation as the sperm swim); beat cross-frequency (BCF, Hz; the frequency of sperm head crossing the average path in either direction); wobble (WOB; VAP/VCL × 100, %; a measure of the oscillation of the actual trajectory about its spatial average path).

### Acrosome integrity

Acrosome integrity was evaluated by incubating spermatozoa with fluorescein isothiocynatelabeled PisumSativum agglutinin (FITC-PSA). The aliquots of sperm suspension were incubated for 15 min at 39 °C with FITC-PSA (5 μg/mL in phosphate buffered saline [PBS], pH 7.4), and propidium iodide (PI; 14 μg/mL in phosphate buffered saline [PBS], pH 7.4). In order to reduce background fluorescence, unbound PSA and PI were removed by adding 200 μL of PBS and spermatozoa were washed by centrifugation in a micro centrifuge at 800 g for 2 min. The supernatant was aspirated and the pellet re-suspended in 100 μL of PBS. After washing, a 10 μL sample was put on a slide and cover slipped. The slide was immediately dried by leaving at 37 °C for 10 min for immobilization of sperm cells. To evaluate the stained sperm cells, at least 200 cells were counted in duplicate for each sample, using a Diaphot (Nikon, Japan) epifluorescence microscope. Spermatozoa with intact plasma membrane and intact acrosome were PI and FITC-PSA negative (no fluorescent staining), those with intact plasma membrane and damaged acrosome were PI negative and FITC-PSA positive (emitting green fluorescence), those with damaged plasma membrane and intact acrosome were PI positive and FITC-PSA negative (emitting red fluorescence), and finally those with damaged plasma membrane and damaged acrosome were PI and FITC-PSA positive (emitting both green and red fluorescence).

### DNA integrity assessment

DNA damage was assessed by single-cell gel electrophoresis (comet assay). Analysis of the shape and length of "comet" tail, just like the DNA content in the tail, gives an assessment of DNA damage. The neutral comet assay allows the detection of double-strand breaks by subjecting lysed cell nuclei to an electrophoretic field at neutral pH [26], here performed according to the method described by Sakkas et al.[27], with slight modifications. Briefly, sperm suspension (30 μL) was diluted in low-melting-point agarose at 37 °C (80 μL; 1% w/v). A 100-μL mixture of sperm-agarose was immediately pipetted onto 1% w/v normal-melting-point agarose-coated slides. Slides were immersed in ice-cold lysing solution (2.5 M NaCl, 100 mM EDTA, 10 mMTris, 1% Triton X, and 10 mMdithiothreitol [DTT]; pH = 10) for 1 h at 4 °C. Slides were then immersed in lysing solution supplemented with proteinase K (10 μg/mL). Incubation was performed during 1 h at 37 °C. After this step, slides were rinsed in PBS and then placed in a horizontal electrophoresis tank filled with freshly prepared electrophoresis neutral buffer (Tris-acetate-EDTA [TAE], pH 7.3). Electrophoresis was performed at 10 V and 6 mA for 20 min. Following electrophoresis, the slides were neutralized with Tris–HCl buffer (pH 7.5) for 5 min and then fixed in methanol.

Slides were stained with propidium iodide (PI), mounted with a coverslip and analyzed under an epifluorescence microscope. Digital comet images were captured with an Olympus microscope equipped with a CCD camera and Olympus CellF software. Fifty comets were measured per replicate sample (i.e., slide circle) using Comet Score software (TriTek Corp., Sumerduck, Virginia, USA). Scored parameters included percentages of head and tail DNA (a measurement of the proportion of total DNA that is present in the comet head and tail).

### Sample preparation for the oxidative parameters analysis

Five mL of semen samples (50×10^6^ spermatozoa/mL) was centrifuged at 1500 g for 10 min. The obtained pellets were treated for cellular extraction with PBS containing 0,1% Triton X-100 (500 μL of PBS-Triton X-100 0,1% every 250×10^6^ total spermatozoa). Malondialdehyde concentration (MDA) and superoxide dismutase (SOD) activity were assayed in cellular extracts, while trolox equivalent antioxidant capacity (TEAC) was determined in both cellular extracts and extracellular supernatants.

### Superoxide dismutase (SOD) activity

SOD activity was measured enzymatically as a decrease of the XTT (3'-(1-[(Phenylamino)-carbonyl]-3,4-tetrazolium)-bis(4-methoxy-6-nitro) benzenesulphonic acid hydrate) reduction by superoxide anion generated by xanthine oxidase [28].

- $$\mathrm{Xanthine}+{\mathrm{O}}_2\overset{\mathrm{XO}}{\to}\mathrm{uric}\;\mathrm{acid}+{{\mathrm{O}}_2}^{-}$$
- $${{\mathrm{O}}_2}^{\hbox{-} }+\mathrm{X}\mathrm{T}\mathrm{T}\;\left(\mathrm{detector}\right)\to \mathrm{reduced}\;\mathrm{X}\mathrm{T}\mathrm{T}$$
- $${{2\mathrm{O}}_2}^{\hbox{-} }+2{\mathrm{H}}^{+}\overset{\mathrm{SOD}}{\to }{\mathrm{H}}_2{\mathrm{O}}_2+{\mathrm{O}}_2$$

SOD activity was assessed as the competition between reaction c and b which is measured as a decrease of the rate of XTT reduced. The reaction mixture contained 40.5 mM sodium phosphate buffer pH 7.8; 15 mM xanthine; EDTA 12,5 mM; XTT 30 mM and 50 μL of sample to complete a final volume of 500 μL. The reaction was initiated by the addition of xantine oxidase (XO) (0.15 mUI) and the absorbance change at 470 nm was monitored each minute for 3 min total with a Hitachi spectrophotometer (U-2000). The values of SOD in the samples were expressed in U/mL and calculated using a standard curve (0,065-0,8 U/ml). One enzyme unit (IU) is defined as the amount of SOD capable of transforming 1.0 mmole/min of O2•¬

### Quantification of lipid peroxidation end products: malondialdehyde (MDA)

MDA, one of the several low-molecular-weight end-products of LPO, was evaluated by the TBARS assay using thiobarbituric acid and a spectrophotometric method according to the TBA test described by Spanier and Traylor [29], with some modifications. 100 μL of each sample (cell extract and extracellular supernatant) were added to 100 μL glacial acetic acid 33%, 75 μL SDS 10%, 100 μL Tris–HCl 50 mM pH 7,4 and 250 μL TBA 0,75%. The mixture was then incubated for 1 h at 100 °C and immediately cooled on ice. After 10 min 200 μL of acetic acid 33% were added and samples were centrifuged for 20 min at 7000 g. The supernatant absorbance was then read with Thermo Electron Corporation Genesys 10UV spectrophotometer (Thermo Fisher Scientific, Rodano, Milano, Italy), at 535 nm. The values of MDA in the samples were expressed in μM units and calculated using a standard curve.

### Trolox equivalent antioxidant capacity (TEAC)

Cell extract and extracellular supernatant antioxidant capacity was determined using the method described by Re et al.[30], and modified by Lewinska et al.[31]. Briefly, a fresh solution was prepared by dissolving 19.5 mg 2,20-azinobis (3- ethylbenzthiazoline −6-sulphonic acid [ABTS]) and 3.3 mg potassium persulphate in 7 mL of 0.1 M phosphate buffer, pH 7.4. This solution was stored in the dark for 12 h for completion of the reaction. ABTS solution was diluted (usually approximately 1:80) in 0.1 mol/L phosphate buffer, pH 7.4, to give an absorbance reading at 734 nm of 1.0. The absorbance of the mixture was measured twice in a spectrophotometer (ThermoElecrom Corporation Genesys 10 UV, Madison, Wisconsin, USA), at 734 nm, 3 min after mixing a sample with the ABTS•¬˙ solution. The extent of ABTS•¬˙ bleaching is proportional to the activity of antioxidants in a given sample. The antioxidant capacity was expressed as TEAC, the concentration of trolox producing the same effect as the sample studied.

The values of TEAC in the samples were calculated using a standard curve (5–20 mMtrolox in a total volume of 550 mL) and were expressed as mMTrolox equivalent for extracellular supernatant and nmoli of trolox equivalent/109 spermatozoa for cell extract.

### Statistical analyses

Statistical analyses were performed using the statistical software program Statgraphic Centurion XV (version15.2.06 for Windows; Stat Point Technologies Inc., Warrenton, VA, USA), and a probability of *p* < 0.05 was considered to be the minimum level of significance. Data are expressed as mean ± S.E. Differences in sperm parameters between the experimental groups at the different time points (hours of treatment) were assessed by general lineal model where: Y = μ + hours of treatment + group + hours of treatment x group + stallion. Hours of treatment and group were considered fixed factors and stallion a random factor. The method used to discriminate between the means was Fisher’s least-significant-difference (l.s.d.) procedure. The probabilities obtained by the l.s.d. test were corrected by Bonferroni’s correction for multiple comparisons. Data were normally distributed (Shapiro Wilk W test: *P* >0.05).

## Results

### Viability

**Fig. 1:**
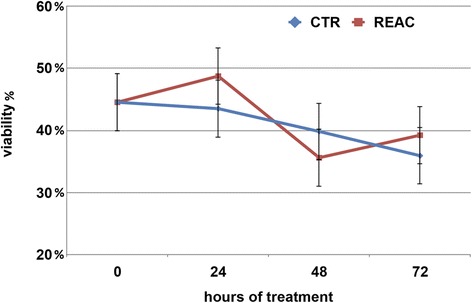
Effect of REAC treatment on stallion spermatozoa viability, expressed expressed as percentage of viable cells over the total cells counted during 72 h. of storage at 4 °C. Values determined at the different time points are expressed as mean ± S.E. A total of 16 ejaculates collected from eight stallions of different breeds (1: Thoroughbred; 2, 3, 4, 6: Arabian; 5, 7, 8: Warmblood) were used

The storage conditions under which sperm cells were maintained proved to preserve spermatozoa viability for at least 72 h. No difference was observed in the number of vital spermatozoa assed by eosin dye exclusion in REAC treated samples and untreated controls (Fig. 1). The male factor was indeed the only factor able to influence spermatozoa viability, as outlined by the significant difference observed among the eight stallions (Additional file 1: Figure S1, panel B).

### Kinetic parameters and straightness index

**Fig. 2:**
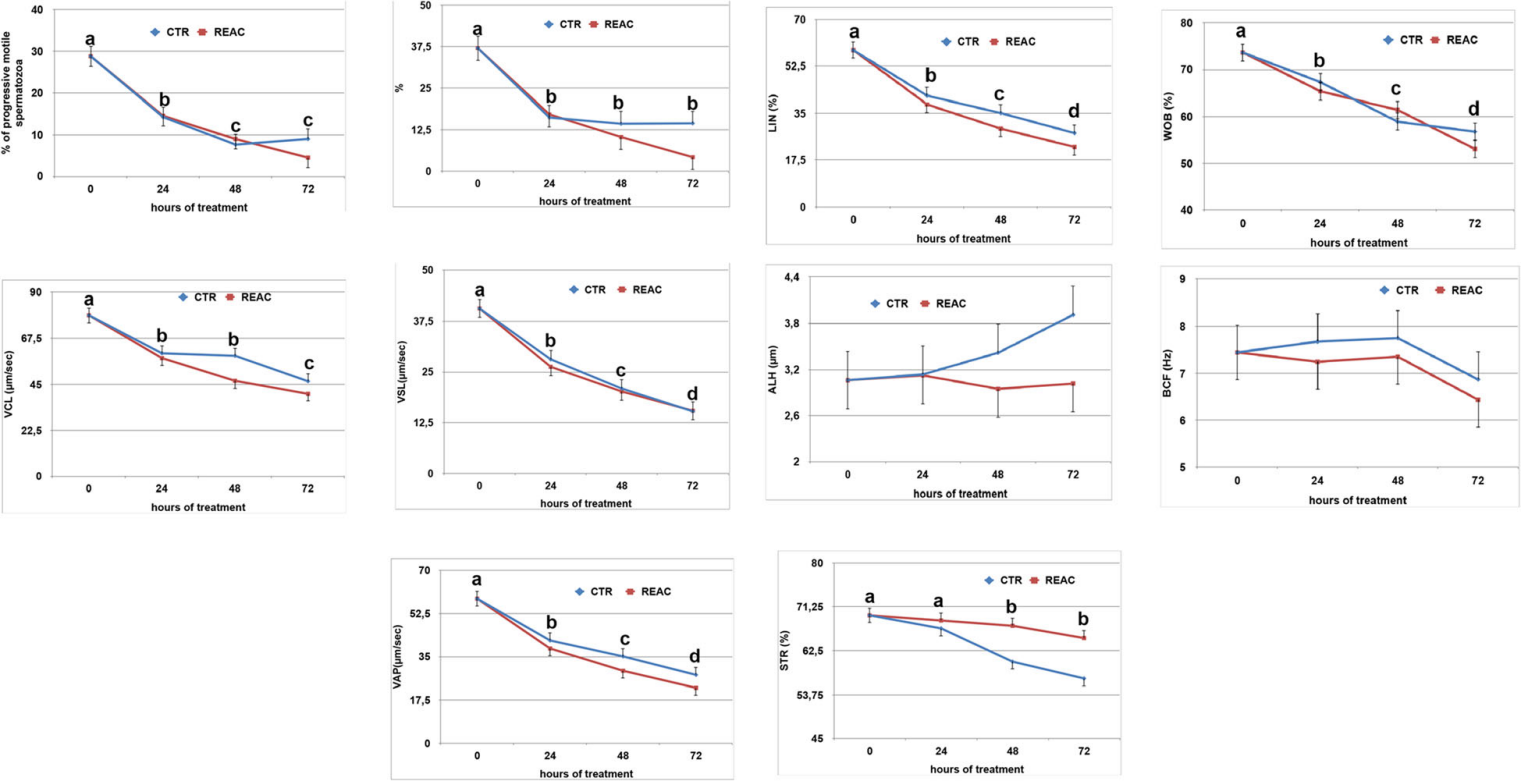
Effect of REAC treatment on the kinetic parameters of stallion spermatozoa during 72 h. of storage at 4 °C. Values determined at the different time points are expressed as mean ± S.E. A total of 16 ejaculates collected from eight stallions of different breeds (1: Thoroughbred; 2, 3, 4, 6: Arabian; 5, 7, 8: Warmblood) were used. Different letters indicate a statistical difference among values recorded at the different time points within the same experimental group (General Linear Model): *p* > 0.01. Upper case letters: REAC group; lower case letters: control group. PM: percentage of progressive motile spermatozoa; Rapid: percentage of rapid spermatozoa; VAP: average path velocity (mm/s; the average velocity of the smoothed cell path); VCL: curvilinear velocity (mm/s; the average velocity measured over the actual point to point track followed by the cell); VSL: straight-line velocity (mm/s; the average velocity measured in a straight line from the beginning to the end of the track); LIN: linearity index (%; the average value of the ratio VSL/VCL); STR: straightness index (%; the average value of the ratio VSL/VAP); ALH: amplitude of lateral head displacement (mm; the mean width of the head oscillation as the sperm swim); BCF: beat cross-frequency (Hz; the frequency of sperm head crossing the average path in either direction); WOB: wobble (VAP/VCL × 100, %; a measure of the oscillation of the actual trajectory about its spatial average path)

In general, kinetic parameters decreased significantly during the 72 h. of observation, and no significant differences were observed between REAC treated samples and untreated controls (Fig. **2**). Interestingly, the straightness index values were significantly higher in REAC treated samples compared to untreated controls (Additional file 2: Figure S2, panel A and B; *p* < 0.001).

### Acrosome membrane integrity

**Fig. 3:**
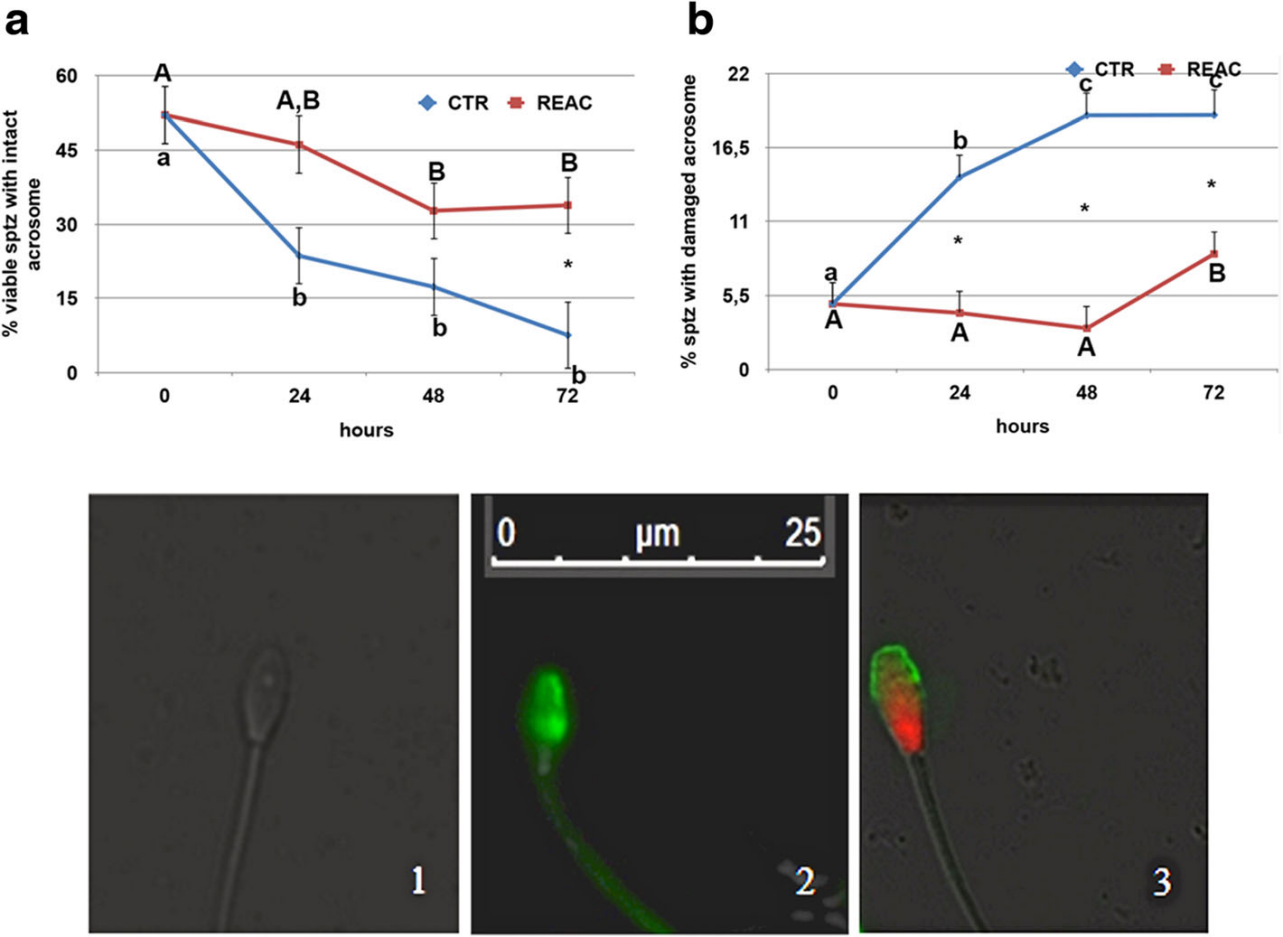
Effect of REAC treatment on acrosome integrity, as evaluated by FITC-PSA staining, of stallion spermatozoa during 72 h. of storage at 4 °C. Panels **a** and **b** show the mean ± S.E values determined at the different time points. Panel A shows the percentage of spermatozoa with intact acrosome, while panel **b** shows the percentage of spermatozoa with damaged acrosome. A total of 16 ejaculates collected from eight stallions of different breeds (1: Thoroughbred; 2, 3, 4, 6: Arabian; 5, 7, 8: Warmblood) were used. The microscopic images show a live spermatozoon with intact acrosome (1; no fluorescent staining), a live spermatozoon with damaged acrosome (2; FITC-PSA positive), and a dead spermatozoon with damaged acrosome (3; PI and FITC-PSA positive), identified using fluorescein isothiocynatelabeledPisumSativum agglutinin (FITC-PSA) and propidium iodide (PI). *Asterisks* indicate statistical differences between REAC treated and untreated controls (General Linear Model): *p* > 0.001. *Different letters* indicate a statistical difference among values recorded at the different time points within the same experimental group (General Linear Model): *p* > 0.01. *Upper case letters*: REAC group; *lower case letters*: control group

Acrosome membrane integrity was better preserved in REAC treated spermatozoa compared to untreated controls, as demonstrated by the higher percentage of spermatozoa showing an intact acrosome after FITC-PSA staining, and by the corresponding lower percentage of spermatozoa with damaged acrosome (*p* < 0.001; Additional file 3: Figure S3, panels A and D). This difference was observed both in the mean values, and in values recorded after 48 and 72 h. of cold storage (Fig. 3). As for the other parameters, acrosome integrity decreased over time with a similar pattern in both experimental groups. In this case, however, individual differences among the eight stallions did not reach statistical significance.

### DNA integrity assessment

**Fig. 4:**
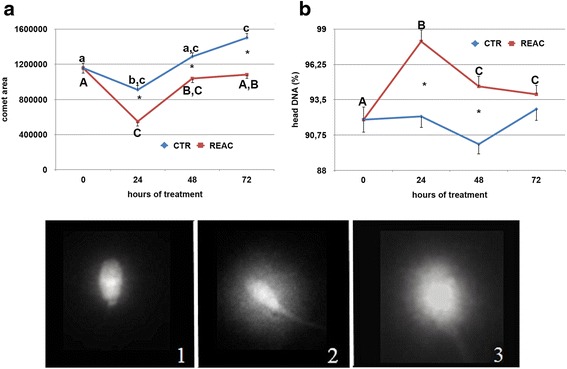
Effect of REAC treatment on DNA integrity, as evaluated by the neutral comet assay, of stallion spermatozoa during 72 h. of storage at 4 °C. Evaluated parameters included the comet area (pixels) and the percentage of DNA in the head. For each parameter, panels **a** and **b** show the mean ± S.E values determined at the different time points in each experimental group. A total of 16 ejaculates collected from eight stallions of different breeds (1: Thoroughbred; 2, 3, 4, 6: Arabian; 5, 7, 8: Warmblood) were used. Images show representative comets following application of the neutral comet assay: (1) non-fragmented sperm nucleus; (2–3) fragmented sperm nuclei. *Asterisks* indicate statistical differences between REAC treated and untreated controls (General Linear Model): *p* > 0.01. *Different letters* indicate a statistical difference among values recorded at the different time points within the same experimental group (General Linear Model): *p* > 0.01. *Upper case letters*: REAC group; *lower case letters*: control group

REAC treatment had a beneficial effect in maintaining spermatozoa DNA integrity, as evaluated by the comet assay (Fig. 4), and this protective effect was evidenced after 24 h. of REAC treatment. A significantly smaller comet area (Additional file 4: Figure S4, panel A; *p* < 0.0001), together with a higher percentage of DNA in the head of the spermatozoa (Additional file 4: Figure S4, panel B; *p* < 0.0001), was found in REAC treated spermatozoa compared to untreated controls. Significant individual differences were observed among the eight stallions in both comet area and percentage of DNA in the head (Additional file 4: Figure S4, panels C and D; *p* > 0.01).

### Oxidative status analysis

**Fig. 5:**
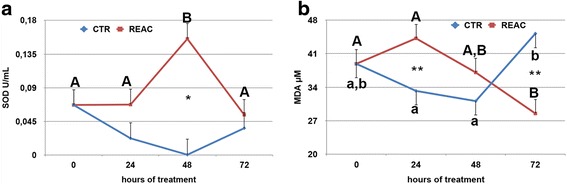
Effect of REAC treatment on SOD (panel **a**) and MDA (panel **b**) intracellular concentrations of stallion spermatozoa during 72 h. of storage at 4 °C. For each parameter, the mean ± S.E values determined at the different time points are shown. A total of 16 ejaculates collected from eight stallions of different breeds (1: Thoroughbred; 2, 3, 4, 6: Arabian; 5, 7, 8: Warmblood) were used. *Asterisks* indicate statistical differences between REAC treated and untreated controls (General Linear Model): * *p* > 0.05; ** *p* < 0.001. *Different letters* indicate a statistical difference among values recorded at different time points within the same experimental group (General Linear Model): *p* > 0.01. *Upper case letters*: REAC group; *lower case letters*: control group

**Fig. 6:**
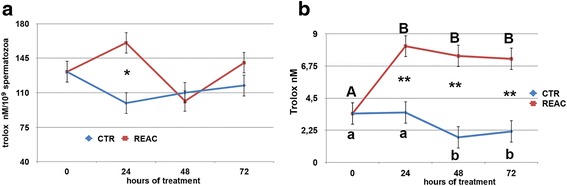
Effect of REAC treatment on TEAC (trolox equivalent antioxidant capacity) in the cellular extract of stallion spermatozoa (panel **a**) and in the media (panel **b**) during 72 h. of storage at 4 °C. For each parameter, panels **a** and **b** show the mean ± S.E values determined at the different time points. A total of 16 ejaculates collected from eight stallions of different breeds (1: Thoroughbred; 2, 3, 4, 6: Arabian; 5, 7, 8: Warmblood) were used. *Asterisks* indicate statistical differences between REAC treated and untreated controls (General Linear Model): * *p* > 0.05; ** *p* < 0.001. Panel **b** *Different letters* indicate a statistical difference among values recorded at the different time points within the same experimental group (General Linear Model): *p* < 0.001. *Upper case letters*: REAC group; *lower case letters*: control group

The analysis of the oxidative status during the 72 h. of cold storage indicated that REAC treatment had a positive effect on the enzymatic defense system of stallion spermatozoa. The mean activity of the intracellular SOD was significantly higher in REAC treated spermatozoa compared to untreated controls (Additional file 5: Figure S5, panel A; *p* < 0.05). This difference was likely linked to the marked increase in the enzyme activity observed after 48 h. of treatment in REAC treated spermatozoa compared to untreated controls (Fig. 5, panel A; *p* < 0.05). The increase in the intracellular antioxidant defenses of the REAC treated spermatozoa was indirectly confirmed by the significant decreased in the intracellular concentration of malondialdehyde (MDA), an end product of lipid peroxidation. While mean values did not differ between the two experimental groups, in REAC treated spermatozoa the intracellular concentration of MDA, after an initial rise at 24 h., dropped to values significantly lower compared to the untreated controls by 72 h. of storage (Fig. 5, panels B and F; *p* < 0.001). Both SOD and MDA intracellular concentration differed significantly among the eight stallions studied. The positive effect of REAC treatment on spermatozoa oxidative status was also supported by the higher trolox equivalent antioxidant capacity (TEAC) found in both the cellular extract (Additional file 6: Figure S6, panel A; *p* < 0.05) and the storage media of REAC treated spermatozoa compared to untreated controls (Additional file 6: Figure S6, panel B; *p* < 0.0001). The antioxidant capacity of the storage media rose significantly during the first 24 h. of REAC treatment, and then remained at higher values compared to untreated controls for the entire observational period (Fig. 6, panel B; *p* < 0.001). The stallion effect influenced significantly only the TEAC values of the cellular extract (Additional file 6: Figure S6, panel C; *p* > 0.0001), while it was not observed for values recorded in the storage media (Additional file 6: Figure S6, panel D, Additional file 7).

## Discussion

The kinetic parameter significantly affected by REAC treatment was spermatozoa straightness index, i.e. the average value of the ratio straight-line velocity/average path velocity, which proved to be better preserved following REAC treatment. This parameter expresses the finalization of spermatozoa towards their fertilization task. In fact, sperm motility is essential for normal fertilization, but the orientation of motility and the motor strategy of spermatozoa are crucial features.

Among the cellular features analyzed, acrosome integrity proved to be significantly influenced by REAC treatment. The mean percentage of spermatozoa with intact acrosome was higher in REAC treated spermatozoa compared with untreated controls. Analyzing the variation of this parameter during the 72 h. of storage, emerged that REAC treatment was effective after 48 h. of storage, as evidenced by the higher percentages of spermatozoa with intact acrosome at 48 and 72 h. Considering that no differences were found in semen viability during storage, the percentage of spermatozoa with damaged acrosome was higher in untreated samples compared with REAC treated ones. The acrosome is necessary for the fertilization process to occur, and a damaged acrosome results inevitably with the loss of the fertilizing ability. The preservation of acrosome membrane integrity in REAC treated spermatozoa was also accompanied by a higher DNA integrity, as revealed by the lower DNA migration after single cell electrophoresis. Even if this parameter was subjected to high inter-individual variations, comet area was significantly larger in untreated controls, the percentage of migrating DNA being higher than in REAC treated spermatozoa. Analyzing the changes in these parameters during storage revealed that REAC beneficial effect on DNA integrity became evident after 24 h. of REAC treatment.

DNA damage in refrigerated cells may be caused by an increase in intracellular sodium concentration, which is detrimental to the nucleus and DNA [18, 19], considering that at low temperature the activity of the sodium-potassium-dependent ATPase is impaired and this can result in uptake of sodium ions from extracellular milieu. Thus, in this study we observed the beneficial effect of REAC treatment as cell polarity optimizer on the preservation of spermatozoa DNA integrity, related to its positive regulation of ion flows and cell bioelectric activity.

In addition, the preservation of both acrosome membrane and DNA integrity in REAC treated spermatozoa can be mediated by the observed effect in enhancing sperm antioxidant defenses. Antioxidants play a crucial role in minimizing oxidative damage in the spermatozoa. The metalloproteins SOD are an important antioxidant defense in nearly all cells exposed to oxygen. SOD, together with glutathione (GSH), is one of the most important intracellular scavenger systems spermatozoa have; it reduces the superoxide anion (O_2_-) to hydrogen peroxide (H_2_O_2_) [32]. The results of this study evidenced that SOD activity in REAC treated spermatozoa peaked at 48 h. of storage and was significantly higher compared to untreated controls. This higher SOD activity was accompanied by a significant decrease over time in intracellular concentration of lipid peroxidase end products (MDA), whose levels at 72 h. of storage were lower in REAC treated spermatozoa compared with untreated controls. In addition, trolox equivalent antioxidant capacity (TEAC) of both cellular extract and storage media was significantly higher in REAC treated samples compared with untreated controls.

Oxidative stress adversely changes sperm cell functions, by ultimately endangering cell survival, and causes loss of sperm motility, viability and capacity for fertilization [33].

The limited volume and restricted location of their cytoplasmic space place constraints on the availability of intracellular antioxidant enzymes in these cells. In addition, the susceptibility of spermatozoa to oxidative stress is a consequence of the superabundance of polyunsaturated fatty acids in their plasma membrane, whose presence gives the membrane the fluidity and flexibility needed to engage in membrane fusion events associated with the fertilization. Unfortunately, the presence of double bonds in these molecules makes them vulnerable to free radical attacks and the initiation of lipid peroxidation (LPO) cascade. These attacks ultimately lead to the impairment of sperm function through oxidative stress and the production of cytotoxic aldehydes, such as MDA [34]. ROS can also attack another important substrate in mammalian spermatozoa: the DNA present in the sperm nucleus [35]. Experimental studies revealed that oxidative stress results in DNA strand breaks in spermatozoa, as detected by the comet assay [36, 37]. Even if it is tightly compacted with protamines, and further stabilized by the creation of inter- and intra-molecular disulphide bonds [36, 38], free radicals can still attack it, engaging in H-abstraction reactions with the ribose unit and inducing the formation of DNA base adducts. Both of these processes greatly destabilize the DNA structure and ultimately result in the formation of DNA strand breaks [35].

For these reasons, oxidative stress of spermatozoa is a main factor in the determination of male infertility. Electron leakage from the sperm mitochondria is thought to constitute the major source of reactive oxygen species (ROS) in spermatozoa [21]. A perturbation in the electron transport chain and the loss of membrane potential are associated with a significant increase in ROS generation [21, 22]. Mitochondria have been identified as the most sensitive sperm structure to cryopreservation [39], and changes in mitochondria membrane potential have been observed in stallion spermatozoa after cooling to 4 °C [10]. The exact mechanisms by which REAC treatment affected spermatozoa oxidative status still have to be elucidated. REAC treatment creates an asymmetric electric circuit in treated cells, which can modulate ion flows when these are altered and which proved to exert a positive regulation of cell bioelectric activity. Thus, we can speculate that the enhancement of sperm antioxidant defenses in REAC treated samples may be mediated by a protective effect on the preservation of mitochondria membrane potential.

## Conclusion

The present study demonstrated that REAC treatment, while not affecting sperm viability and motility during refrigeration, preserves spermatozoa acrosome membrane and DNA integrity. This effect is likely to be mediated by the enhancement of sperm antioxidant defenses after REAC treatment, as evidenced by the higher SOD activity, the lower MDA intracellular concentration at the end of the treatment, and the higher antioxidant capacity of the cell extract and the culture medium. As suggested by other studies, any mean aimed at enhancing sperm antioxidant defenses has an important therapeutic role to play in the clinical management of male infertility [33]. Thus, this study set the basis for the use of REAC protocols aimed at enhancing the spermatozoa ability to withstand the oxidative stress related with Assisted Reproductive Technology (ART) processing.

The present study demonstrated that REAC treatment during liquid storage of chilled stallion spermatozoa preserves sperm antioxidant defenses, and as a result allows acrosome membrane and DNA integrity to be maintained longer than in untreated controls. While the loss of acrosome membrane integrity results in an impairment in spermatozoa fertilizing ability, DNA damage in the male germ line is a major contributor to infertility, miscarriage and birth defects in the offspring [40]. Thus, this technology has promising application in the in vitro treatment of sperm cells during ART processing.

## Additional files

Additional file 1: Figure S1. Mean and 95,0 Bonferroni intervals of stallion spermatozoa viability, expressed as percentage of viable cells over the total cells counted. Values refer to the mean of the different values per group (control vs REAC treated, panel A) and per stallion (panel B) obtained during 72 h. of storage at 4°C. A total of 16 ejaculates collected from 8 stallions of different breeds (1: Thoroughbred; 2, 3, 4, 6: Arabian; 5, 7, 8: Warmblood) were used. ^a,b,c,d^ Different letters indicate a statistical difference among the ejaculates collected from the 8 stallions (General Linear Model): p>0.01. (TIF 332 kb)
Additional file 2: Figure S2. Effect of REAC treatment on the kinetic parameters of stallion spermatozoa during 72 h. of storage at 4°C. Panel A and B show the mean and 95,0 Bonferroni intervals per group and per stallion, respectively. A total of 16 ejaculates collected from 8 stallions of different breeds (1: Thoroughbred; 2, 3, 4, 6: Arabian; 5, 7, 8: Warmblood) were used. Panel B: ^a,b,c,d^ Different letters indicate a statistical difference among the ejaculates collected from the 8 stallions (General Linear Model): *p*>0.001. PM: percentage of progressive motile spermatozoa; Rapid: percentage of rapid spermatozoa; VAP: average path velocity (mm/s; the average velocity of the smoothed cell path); VCL: curvilinear velocity (mm/s; the average velocity measured over the actual point to point track followed by the cell); VSL: straight-line velocity (mm/s; the average velocity measured in a straight line from the beginning to the end of the track); LIN: linearity index (%; the average value of the ratio VSL/VCL); STR: straightness index (%; the average value of the ratio VSL/VAP); ALH: amplitude of lateral head displacement (mm; the mean width of the head oscillation as the sperm swim); BCF: beat cross-frequency (Hz; the frequency of sperm head crossing the average path in either direction); WOB: wobble (VAP/VCL × 100, %; a measure of the oscillation of the actual trajectory about its spatial average path). (TIF 541 kb)
Additional file 3: Figure S3. Effect of REAC treatment on acrosome integrity, as evaluated by FITC-PSA staining, of stallion spermatozoa during 72 h. of storage at 4 °C. Panels A and D show the mean and 95,0 Bonferroni intervals per group, while panels B and C show them per stallion. Left panels show the percentage of spermatozoa with intact acrosome, while right panels show the percentage of spermatozoa with damaged acrosome. A total of 16 ejaculates collected from 8 stallions of different breeds (1: Thoroughbred; 2, 3, 4, 6: Arabian; 5, 7, 8: Warmblood) were used. Panels A and D: Asterisks indicate statistical differences between REAC treated and untreated controls (General Linear Model): *p*>0.001. (TIF 425 kb)
Additional file 4: Figure S4. Effect of REAC treatment on DNA integrity, as evaluated by the neutral comet assay, of stallion spermatozoa during 72 h. of storage at 4 °C. Evaluated parameters included the comet area (pixels) and the percentage of DNA in the head. For each parameter, panels A and B show the mean and 95,0 Bonferroni intervals per group, while panels C and D show them per stallion. A total of 16 ejaculates collected from 8 stallions of different breeds (1: Thoroughbred; 2, 3, 4, 6: Arabian; 5, 7, 8: Warmblood) were used. Asterisks indicate statistical differences between REAC treated and untreated controls (General Linear Model): *p*>0.01. (TIF 372 kb)
Additional file 5: Figure S5. Effect of REAC treatment on SOD (left side) and MDA (right side) intracellular concentrations of stallion spermatozoa during 72 h. of storage at 4°C. For each parameter, panels A and B show the mean and 95,0 Bonferroni intervals per group, while panels C and D show them per stallion. A total of 16 ejaculates collected from 8 stallions of different breeds (1: Thoroughbred; 2, 3, 4, 6: Arabian; 5, 7, 8: Warmblood) were used. Asterisks indicate statistical differences between REAC treated and untreated controls (General Linear Model): *p*>0.05. Different letters indicate a statistical difference among horses (General Linear Model): *p*>0.01. (TIF 397 kb)
Additional file 6: Figure S6. Effect of REAC treatment on TEAC (trolox equivalent antioxidant capacity) in the cellular extract of stallion spermatozoa (left side) and in the media (right side) during 72 h. of storage at 4 °C. For each parameter, panels A and B show the mean and 95,0 Bonferroni intervals per group, while panels C and D show them per stallion. A total of 16 ejaculates collected from 8 stallions of different breeds (1: Thoroughbred; 2, 3, 4, 6: Arabian; 5, 7, 8: Warmblood) were used. Asterisks indicate statistical differences between REAC treated and untreated controls (General Linear Model): * *p*>0.05; ** *p*<0.0001. Different letters indicate a statistical difference among horses and among values recorded at the different time points within the same experimental group (General Linear Model): Panel C: *p*>0.0001. Panel F: *p*<0.001. Upper case letters: REAC group; lower case letters: control group. (TIF 401 kb)
Additional file 7: Experimental raw data. (XLSX 351 kb)

## Abbreviations

ALH: Lateral head displacement
ART: Assisted Reproductive Technology
BCF: Beat cross-frequency
CASA: Computer-assisted sperm analysis system
FITC-PSA: Fluorescein isothiocynatelabeled PisumSativum agglutinin
GSH: Glutathione
LIN: Linearity index
LPO: Lipid peroxidation
MDA: Malondialdehyde
PBS: Phosphate buffered saline
PI: Propidium iodide
PM: Progressive motile spermatozoa
REAC technology: Radio Electric Asymmetric Conveyor Technology
ROS: Reactive oxygen species
SOD: Superoxide dismutase
STR: Straightness index
TBA: Thiobarbituric acid
TBARS: Thiobarbituric acid reactive substances assay
TBS: Tartrate phosphate buffer
TEAC: Trolox equivalent antioxidant capacity
VAP: Average path velocity
VCL: Curvilinear velocity of spermatozoa
VSL: Straight-line velocity
WOB: Wobble index
