# Supplementary figures and images for: REAC technology as optimizer of stallion spermatozoa liquid storage
Source: Reprod Biol Endocrinol. 2017 Feb 8;15:11. doi: 10.1186/s12958-017-0229-6 (PMC5299698; doi:10.1186/s12958-017-0229-6)

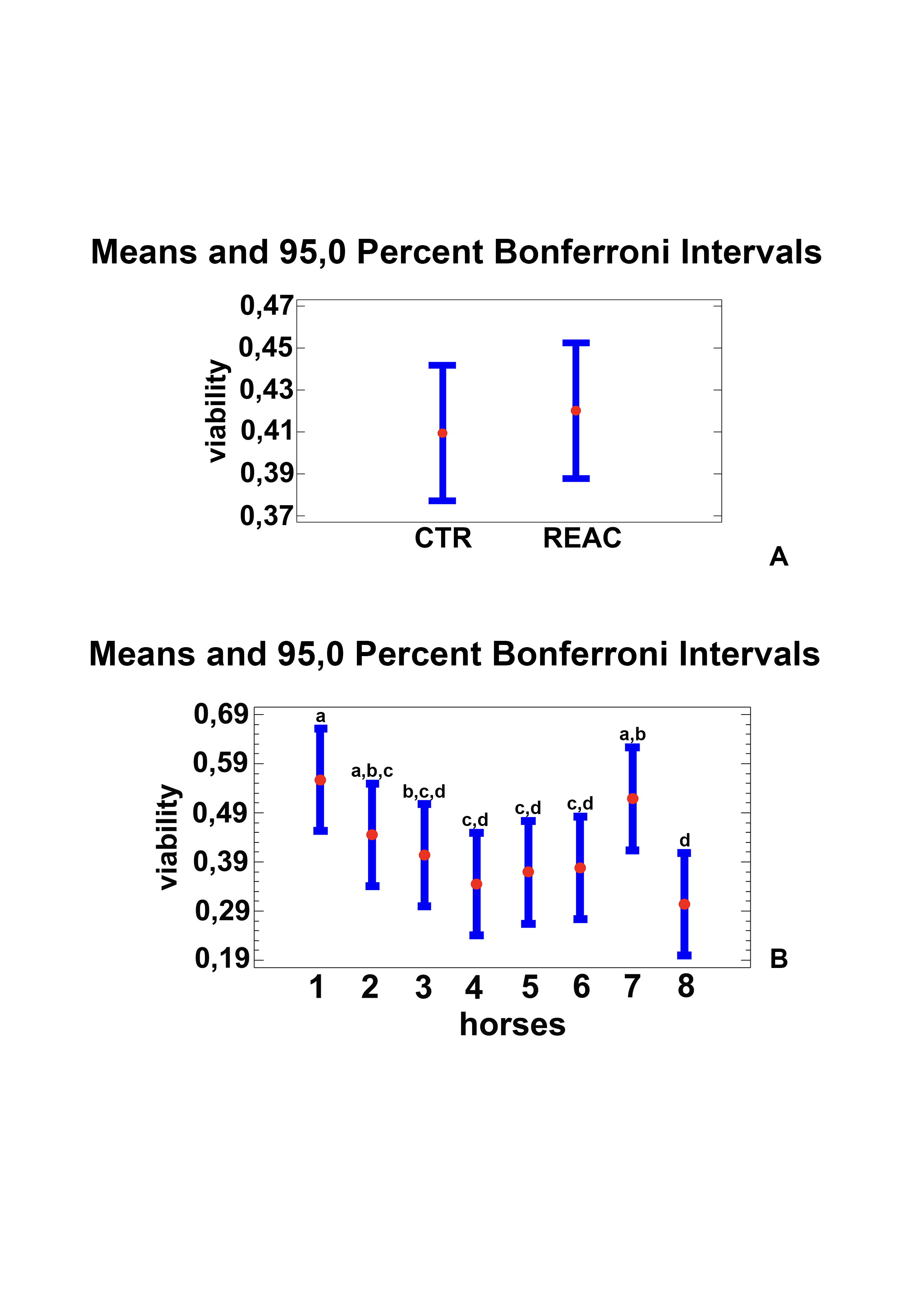

Supplement: Additional file 1: Figure S1. — Mean and 95,0 Bonferroni intervals of stallion spermatozoa viability, expressed as percentage of viable cells over the total cells counted. Values refer to the mean of the different values per group (control vs REAC treated, panel A) and per stallion (panel B) obtained during 72 h. of storage at 4°C. A total of 16 ejaculates collected from 8 stallions of different breeds (1: Thoroughbred; 2, 3, 4, 6: Arabian; 5, 7, 8: Warmblood) were used. a,b,c,d Different letters indicate a statistical difference among the ejaculates collected from the 8 stallions (General Linear Model): p>0.01. (TIF 332 kb) [file 12958_2017_229_MOESM1_ESM.tif]

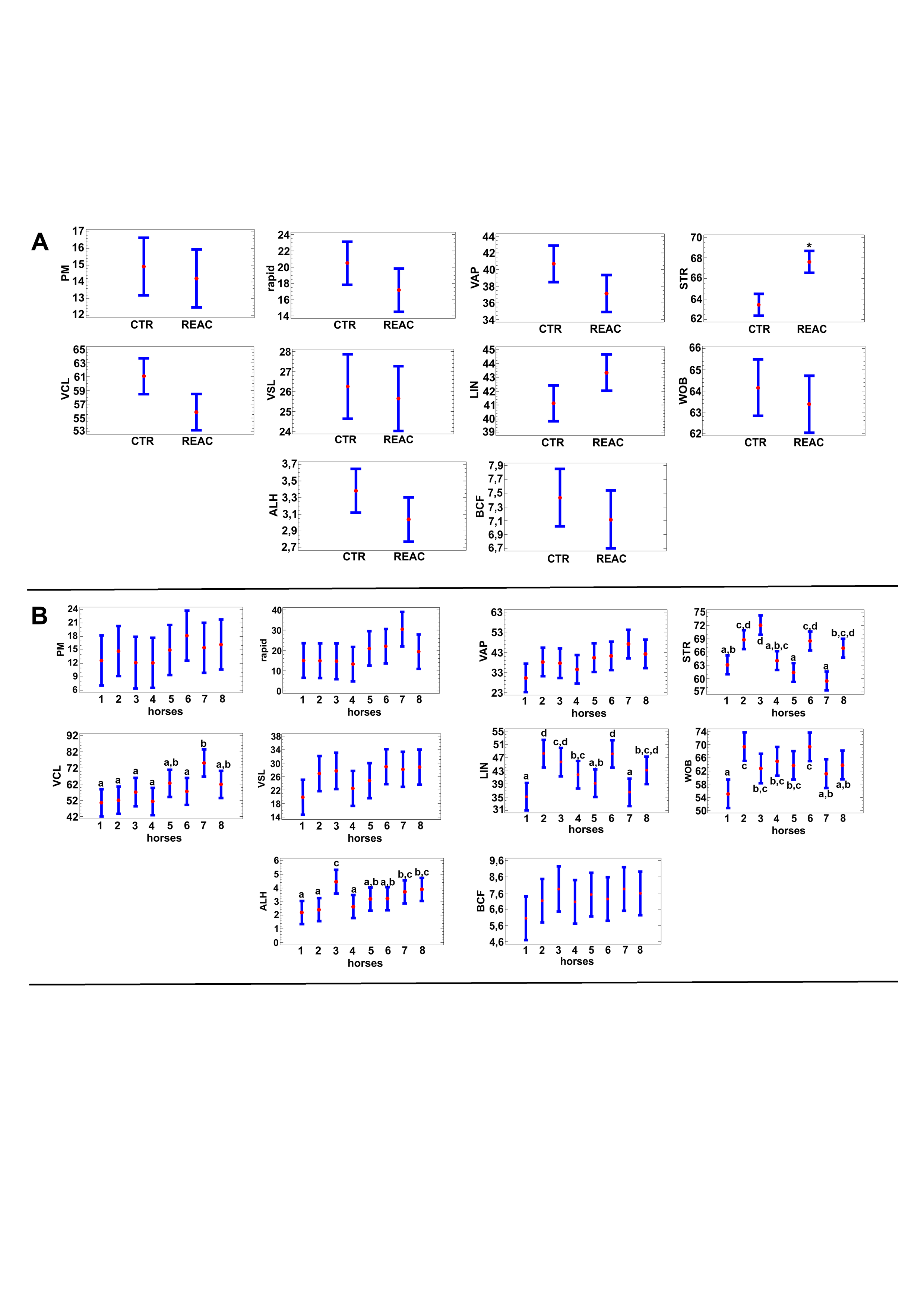

Supplement: Additional file 2: Figure S2. — Effect of REAC treatment on the kinetic parameters of stallion spermatozoa during 72 h. of storage at 4°C. Panel A and B show the mean and 95,0 Bonferroni intervals per group and per stallion, respectively. A total of 16 ejaculates collected from 8 stallions of different breeds (1: Thoroughbred; 2, 3, 4, 6: Arabian; 5, 7, 8: Warmblood) were used. Panel B: a,b,c,d Different letters indicate a statistical difference among the ejaculates collected from the 8 stallions (General Linear Model): p>0.001. PM: percentage of progressive motile spermatozoa; Rapid: percentage of rapid spermatozoa; VAP: average path velocity (mm/s; the average velocity of the smoothed cell path); VCL: curvilinear velocity (mm/s; the average velocity measured over the actual point to point track followed by the cell); VSL: straight-line velocity (mm/s; the average velocity measured in a straight line from the beginning to the end of the track); LIN: linearity index (%; the average value of the ratio VSL/VCL); STR: straightness index (%; the average value of the ratio VSL/VAP); ALH: amplitude of lateral head displacement (mm; the mean width of the head oscillation as the sperm swim); BCF: beat cross-frequency (Hz; the frequency of sperm head crossing the average path in either direction); WOB: wobble (VAP/VCL × 100, %; a measure of the oscillation of the actual trajectory about its spatial average path). (TIF 541 kb) [file 12958_2017_229_MOESM2_ESM.tif]

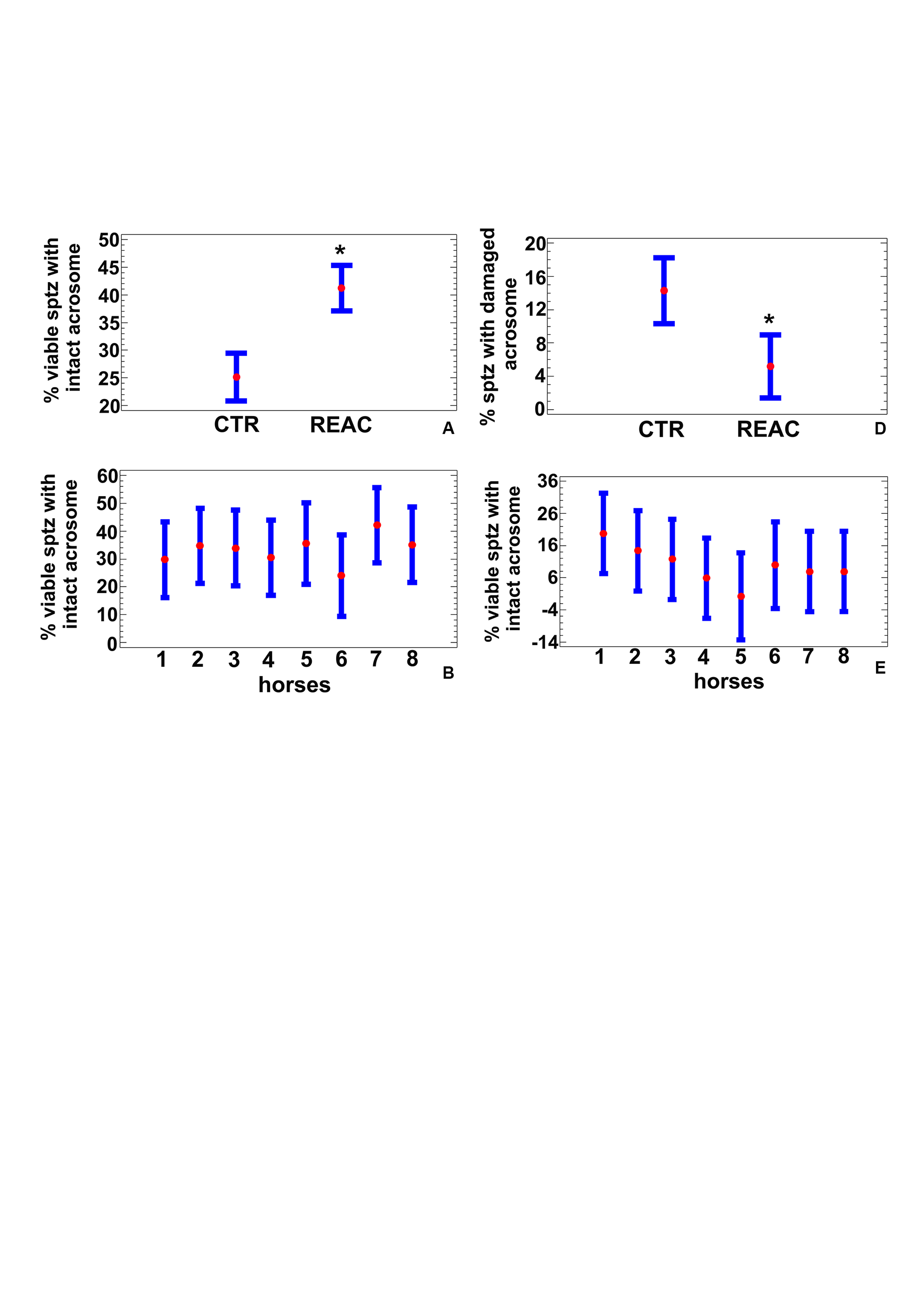

Supplement: Additional file 3: Figure S3. — Effect of REAC treatment on acrosome integrity, as evaluated by FITC-PSA staining, of stallion spermatozoa during 72 h. of storage at 4 °C. Panels A and D show the mean and 95,0 Bonferroni intervals per group, while panels B and C show them per stallion. Left panels show the percentage of spermatozoa with intact acrosome, while right panels show the percentage of spermatozoa with damaged acrosome. A total of 16 ejaculates collected from 8 stallions of different breeds (1: Thoroughbred; 2, 3, 4, 6: Arabian; 5, 7, 8: Warmblood) were used. Panels A and D: Asterisks indicate statistical differences between REAC treated and untreated controls (General Linear Model): p>0.001. (TIF 425 kb) [file 12958_2017_229_MOESM3_ESM.tif]

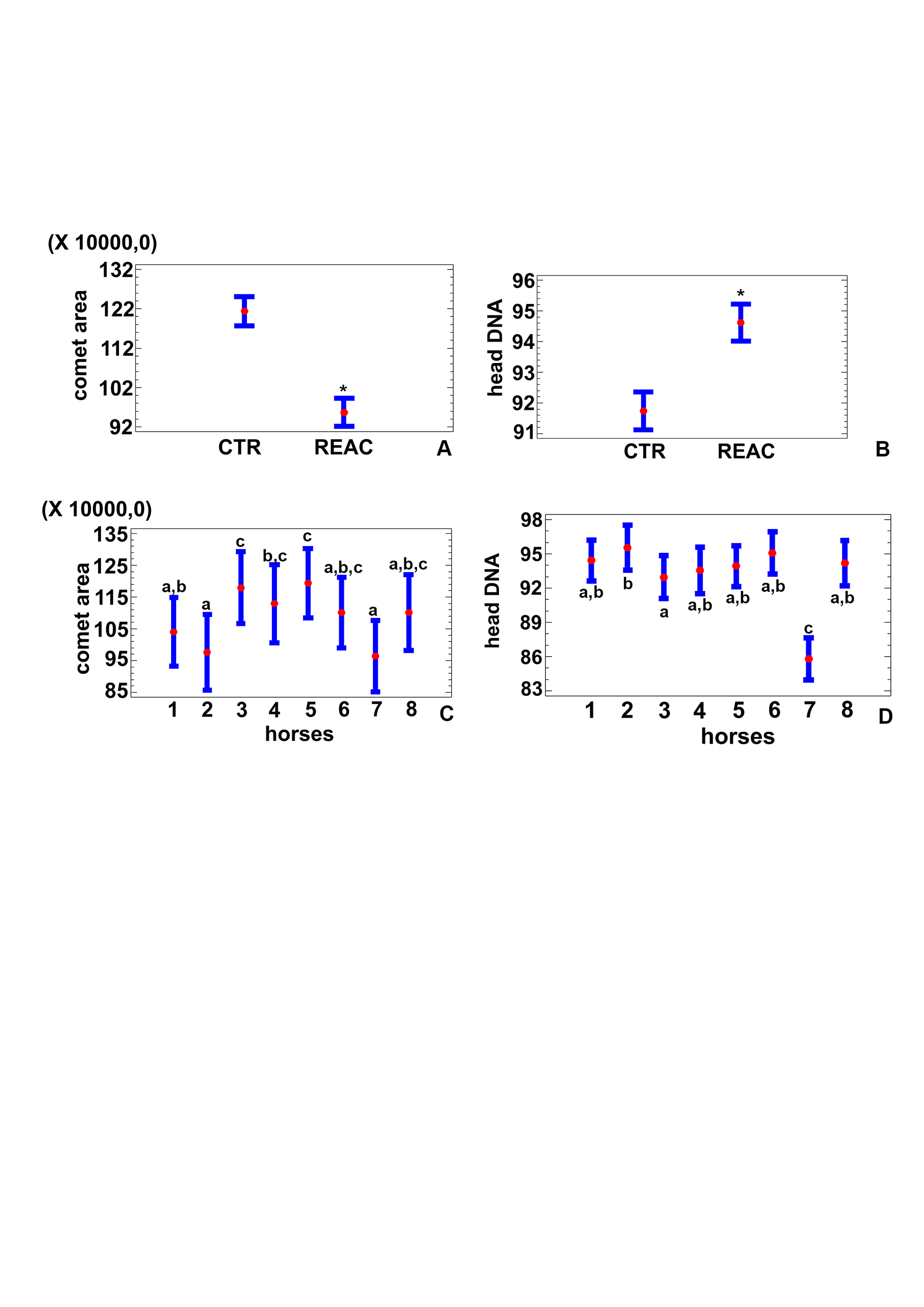

Supplement: Additional file 4: Figure S4. — Effect of REAC treatment on DNA integrity, as evaluated by the neutral comet assay, of stallion spermatozoa during 72 h. of storage at 4 °C. Evaluated parameters included the comet area (pixels) and the percentage of DNA in the head. For each parameter, panels A and B show the mean and 95,0 Bonferroni intervals per group, while panels C and D show them per stallion. A total of 16 ejaculates collected from 8 stallions of different breeds (1: Thoroughbred; 2, 3, 4, 6: Arabian; 5, 7, 8: Warmblood) were used. Asterisks indicate statistical differences between REAC treated and untreated controls (General Linear Model): p>0.01. (TIF 372 kb) [file 12958_2017_229_MOESM4_ESM.tif]

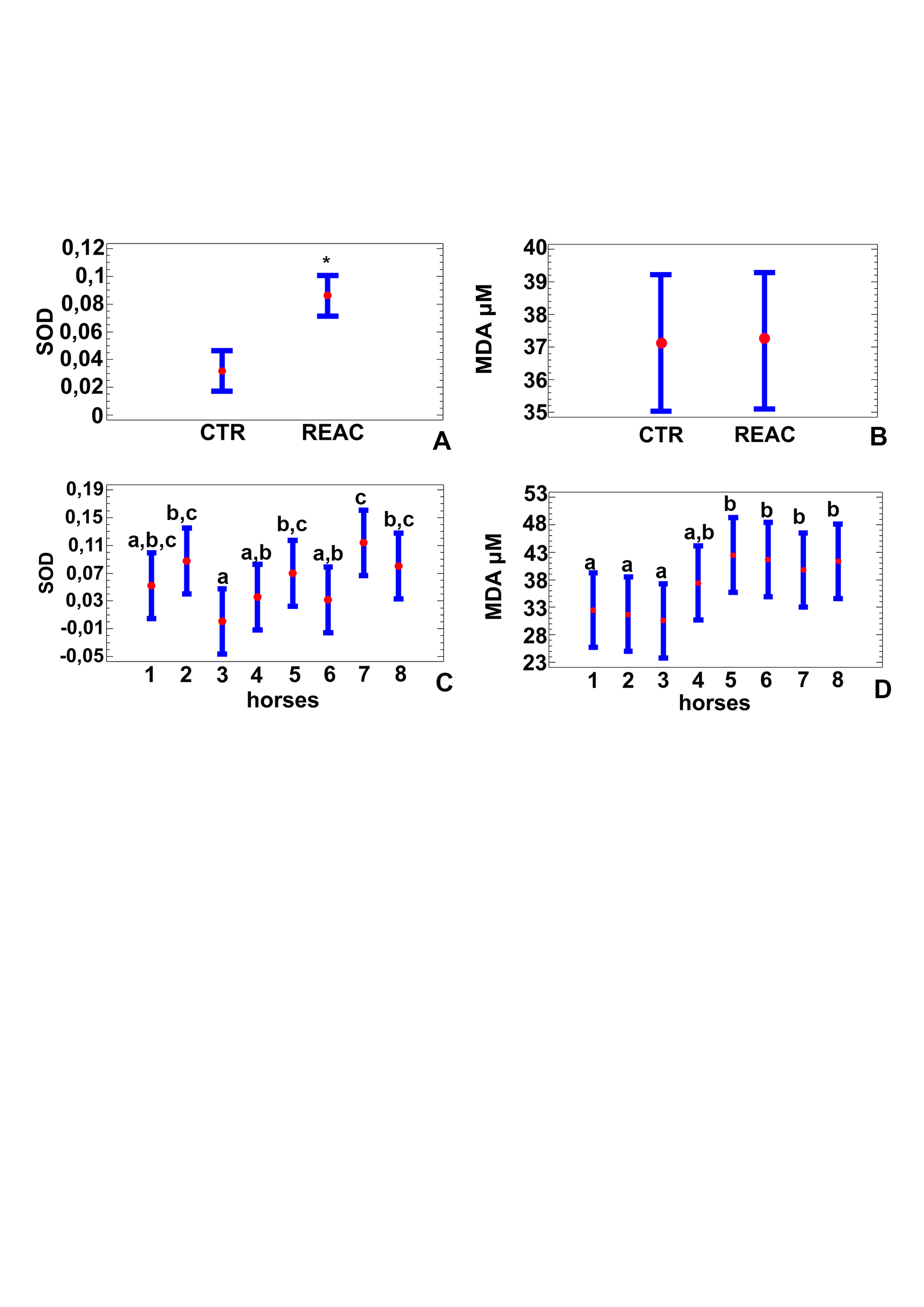

Supplement: Additional file 5: Figure S5. — Effect of REAC treatment on SOD (left side) and MDA (right side) intracellular concentrations of stallion spermatozoa during 72 h. of storage at 4°C. For each parameter, panels A and B show the mean and 95,0 Bonferroni intervals per group, while panels C and D show them per stallion. A total of 16 ejaculates collected from 8 stallions of different breeds (1: Thoroughbred; 2, 3, 4, 6: Arabian; 5, 7, 8: Warmblood) were used. Asterisks indicate statistical differences between REAC treated and untreated controls (General Linear Model): p>0.05. Different letters indicate a statistical difference among horses (General Linear Model): p>0.01. (TIF 397 kb) [file 12958_2017_229_MOESM5_ESM.tif]

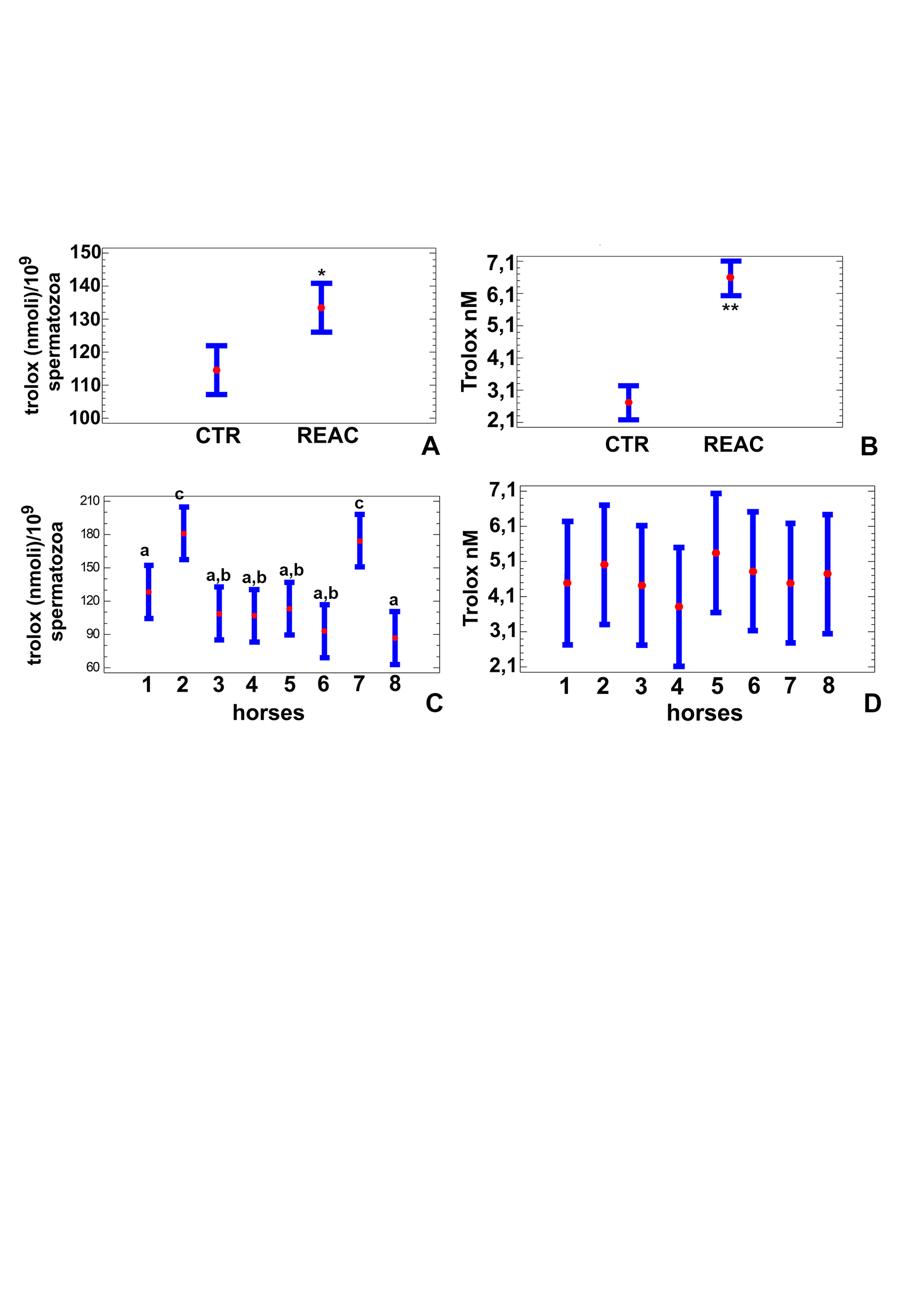

Supplement: Additional file 6: Figure S6. — Effect of REAC treatment on TEAC (trolox equivalent antioxidant capacity) in the cellular extract of stallion spermatozoa (left side) and in the media (right side) during 72 h. of storage at 4 °C. For each parameter, panels A and B show the mean and 95,0 Bonferroni intervals per group, while panels C and D show them per stallion. A total of 16 ejaculates collected from 8 stallions of different breeds (1: Thoroughbred; 2, 3, 4, 6: Arabian; 5, 7, 8: Warmblood) were used. Asterisks indicate statistical differences between REAC treated and untreated controls (General Linear Model): * p>0.05; ** p<0.0001. Different letters indicate a statistical difference among horses and among values recorded at the different time points within the same experimental group (General Linear Model): Panel C: p>0.0001. Panel F: p<0.001. Upper case letters: REAC group; lower case letters: control group. (TIF 401 kb) [file 12958_2017_229_MOESM6_ESM.tif]
